# Mechanical ventilation worsens abdominal edema and inflammation in porcine endotoxemia

**DOI:** 10.1186/cc12801

**Published:** 2013-06-24

**Authors:** Marco Lattuada, Maria Bergquist, Enn Maripuu, Göran Hedenstierna

**Affiliations:** 1 Department of Medical Sciences, Clinical Physiology, Hedenstierna Laboratory, Uppsala University, Sweden; 2 Department of Anesthesia and Intensive Care, Fondazione IRCCS Ca' Granda, Ospedale Maggiore Policlinico, Milan, Italy; 3 Department of Medical Sciences, Clinical Physiology, Hedenstierna Laboratory, Uppsala University, Sweden; 4 Department of Medical Physics, University Hospital, Uppsala, Sweden; 5 Department of Medical Sciences, Clinical Physiology, Hedenstierna Laboratory, Uppsala University, Sweden

## Abstract

**Introduction:**

We hypothesized that mechanical ventilation *per se *increases abdominal edema and inflammation in sepsis and tested this in experimental endotoxemia.

**Methods:**

Thirty anesthetized piglets were allocated to one of five groups: healthy control pigs breathing spontaneously with continuous positive pressure of 5 cm H_2_O or mechanically ventilated with positive end-expiratory pressure of 5 cm H_2_O, and endotoxemic piglets during mechanical ventilation for 2.5 hours and then continued on mechanical ventilation with positive end-expiratory pressure of either 5 or 15 cm H_2_O or switched to spontaneous breathing with continuous positive pressure of 5 cm H_2_O for another 2.5 hours. Abdominal edema formation was estimated by isotope technique, and inflammatory markers were measured in liver, intestine, lung, and plasma.

**Results:**

Healthy controls: 5 hours of spontaneous breathing did not increase abdominal fluid, whereas mechanical ventilation did (Normalized Index increased from 1.0 to 1.6; 1 to 3.3 (median and range, *P *< 0.05)). Endotoxemic animals: Normalized Index increased almost sixfold after 5 hours of mechanical ventilation (5.9; 4.9 to 6.9; *P *< 0.05) with twofold increase from 2.5 to 5 hours whether positive end-expiratory pressure was 5 or 15, but only by 40% with spontaneous breathing (*P *< 0.05 versus positive end-expiratory pressure of 5 or 15 cm H_2_O). Tumor necrosis factor-α (TNF-α) and interleukin (IL)-6 in intestine and liver were 2 to 3 times higher with mechanical ventilation than during spontaneous breathing (*P *< 0.05) but similar in plasma and lung. Abdominal edema formation and TNF-α in intestine correlated inversely with abdominal perfusion pressure.

**Conclusions:**

Mechanical ventilation with positive end-expiratory pressure increases abdominal edema and inflammation in intestine and liver in experimental endotoxemia by increasing systemic capillary leakage and impeding abdominal lymph drainage.

## Introduction

Hospital mortality in sepsis varies between 30% and 75%, with the highest mortality in patients with septic shock [1-5]. The poor outcome is often caused by multiorgan failure, such as intestinal, kidney, and liver failure [6,7]. We previously showed that mechanical ventilation with positive end-expiratory pressure (PEEP) of 5 or 15 cm H_2_O in piglets impedes abdominal lymph flow compared with spontaneous breathing with a continuous positive airway pressure (CPAP) of 5 cm H_2_O [8]. This held true whether the piglets were essentially healthy or exposed to endotoxin infusion to create a sepsis-like condition. Lymphatics play an important role in preventing tissue edema by removing extravascular fluid and proteins. If capillary leakage is increased or drainage is impeded, edema may ensue. We hypothesized that mechanical ventilation in itself promotes abdominal edema by increasing systemic capillary pressure (increased vascular leakage) and by compressing the thoracic duct when airway and intrathoracic pressure are increased (impeded lymph drainage). We therefore studied, in a porcine endotoxin model, abdominal edema formation and inflammatory response in abdominal organs during mechanical ventilation (MV) and spontaneous breathing (SB). To this end, we applied a double isotope gamma camera technique to evaluate edema formation [9] and enzyme-linked immunosorbent assay (ELISA) for assessing inflammatory markers.

## Materials and methods

The study was approved by the Animal Research Ethical Committee of Uppsala University and was performed in accordance with the ethical standards laid down in the 1964 Declaration of Helsinki and its later amendments.

### Animal preparation

Thirty anesthetized and tracheotomized piglets, 2 to 3 months old, with a mean weight (± standard deviation) of 23.2 ± 1.7 kg were studied. The animals were ventilated in volume-controlled mode, respiratory rate of 20 to 22 breaths/minute, tidal volume of 10 ml/kg, inspiration/expiration ratio of 1:2, PEEP of 5 cm H_2_O, and inspired fraction of O_2_, 0.5. The animals were placed supine on a heated table. Peak and mean airway pressures were recorded. The end-inspiratory pressure could not be reliably measured in spontaneously breathing animals with the presently used technique, and data were discarded.

Catheters were introduced into pulmonary and systemic arteries and central veins for pressure recordings, cardiac-output determinations, and blood sampling. A urinary catheter was placed in the bladder for estimating intraabdominal pressure [10].

### Protocol

Healthy control pigs were either breathing spontaneously with CPAP of 5 cm H_2_O (*n *= 6) or mechanically ventilated with PEEP, 5 cm H_2_O (*n *= 6) for 5 hours. Abdominal edema formation was estimated after 5 hours with a double-isotope technique (see later).

**Figure 1:**
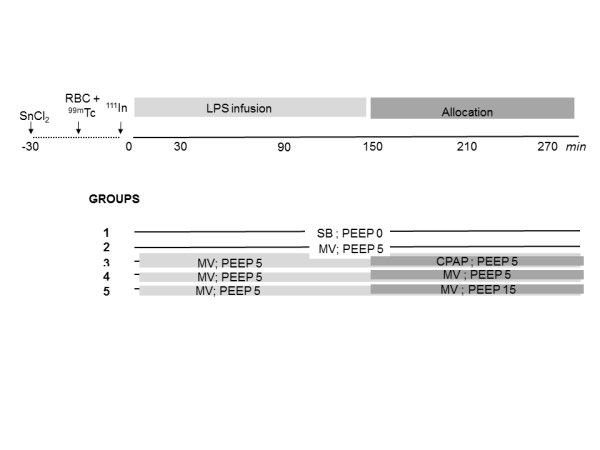
**Schematic drawing of the protocol**. **(Top) **Time-line and essential interventions. **(Bottom) **Group description. SB, spontaneous breathing; CPAP, continuous positive airway pressure; MV, mechanical ventilation; PEEP, positive end-expiratory pressure; LPS, endotoxin infusion. For further details, see text.

Endotoxin pigs (n = 18) were given an intravenous endotoxin infusion (LPS, *Escherichia coli *0111:B4; Sigma Chemicals, St. Louis, MO, USA) at a dose of 15 μg/kg/h for 2.5 hours during MV with PEEP of 5 cm H_2_O MV + PEEP5. They were then either maintained on (a) MV + PEEP5 or switched to (b) MV with PEEP of 15 cm H_2_O or (c) SB with CPAP of 5 cm H_2_O for another 2.5 hours (*n *= 6 in each group). The protocol is schematically given in Figure 1.

At the end of the study, blood was collected, and tissue biopsies were taken for assessing inflammatory markers (TNF-α and IL-6 in plasma and tissue from liver, intestine, and lung, by using enzyme-linked immunosorbent assays (ELISAs). The animals were killed with intravenous KCl.

### Gamma camera measurements

The estimation of abdominal edema with gamma camera measurement has been described in detail previously [9]. A short summary is given here.

An intravascular marker (red blood cells, RBCs, labeled with technetium-99m (^99m^Tc)) and a marker that could leak outside the vasculature (transferrin labeled with indium-111 (^111^In)) were used.

Extravascular fluid and protein accumulation (edema formation) in a selected region of interest (ROI) was assessed by calculating a normalized index (NI):

$$\text{NI}\;\left({\text{t}}_{\text{i}}\right)=\text{I}{\text{n}}_{\text{ROI}}\left({\text{t}}_{\text{i}}\right)\text{/I}{\text{n}}_{\text{ROI}}\left({\text{t}}_{0}\right)\;\text{X}\;\text{T}{\text{c}}_{\text{bl}}\left({\text{t}}_{\text{i}}\right)\text{/T}{\text{c}}_{\text{bl}}\left({\text{t}}_{0}\right)$$

$$\text{I}{\text{n}}_{\text{bl}}\left({\text{t}}_{\text{i}}\right)\text{/I}{\text{n}}_{\text{bl}}\left({\text{t}}_{0}\right)\;\;\;\text{T}{\text{c}}_{\text{ROI}}\left({\text{t}}_{\text{i}}\right)\text{/T}{\text{c}}_{\text{ROI}}\left({\text{t}}_{0}\right)$$

where In_ROI _= transferrin count in the ROI, In_bl _= transferrin count in the blood, Tc_ROI _= RBC count in the ROI, Tc_bl _= RBC count in the blood, t_0 _= time of labeling, and t_i _= time of measurement.

The ROIs were drawn by (a) encompassing the whole abdomen, (b) covering the major part of the liver but with a distance of 1 cm to its border to ensure that the ROI consisted of liver tissue, and (c) the left half of the abdomen, mainly reflecting free fluid (9). ROIs were drawn independently by two investigators, and, if a difference was found, a mean contour of the ROI was applied.

### Statistical analysis

Data are presented as median and range because of the limited number of animals in each group, and nonparametric tests were used for comparisons between groups.

Comparisons were made (a) between healthy control pigs that were breathing spontaneously with CPAP 5 cm H_2_O and healthy control pigs that were mechanically ventilated with PEEP 5 cm H_2_O, and (b) between endotoxin pigs on MV with PEEP 5 cm H_2_O, MV with PEEP 15 cm H_2_O or SB with CPAP 5 cm H_2_O. All group comparisons were made with the Mann-Whitney test. A Bonferroni correction was made for multiple comparisons [11]. All calculations were made with SAS v 9.3.

## Results

### Healthy controls

*Gas exchange and hemodynamics: *The pigs were well oxygenated whether they were breathing spontaneously or were ventilated mechanically, although arterial oxygen tension (PaO_2_) was higher in the MV group. Arterial carbon dioxide tension (PaCO_2_) was within normal range and did not differ between SB and MV pigs. No change was seen over the 5-hour study period (Table 1).

**Table 1:** Ventilation pressures and arterial blood gases at Baseline and at 150 and 300 min (median and range).

	Study group	Baseline All healthy	150 minutes Healthy/LPS	Change to ventilation	300 minutes Healthy/LPS
**P_aw _max** **cm H_2_O**	PEEP 5 Healthy	18.5; 17-21	18.7; 17-22		21.2; 18-26
				CPAP 5 LPS	9.0; 8-10
	PEEP 5 LPS	20.1; 17-23	25.3; 18-35	PEEP 5 LPS	26.5; 25-32^4^
				PEEP 15 LPS	34.0; 32-37^4,5^
**P_aw _mean**	SB Healthy	2,0; 2.0-2.0	1.5; 1.0-3.0		1.0; 1.0-2.0
**cm H_2_O**	PEEP 5 Healthy	9.0; 8.0-10.0^1^	9.0; 8.0-10.0		9.0; 8.0-10.0^1^
				CPAP 5 LPS	6.0; 5.0-6.0
	PEEP 5 LPS	8.3; 8.0-9.0	9.8; 9-11	PEEP 5 LPS	10; 9.0-11.0^4^
				PEEP 15 LPS	19.5; 19.0-20.0,^4,5^
**PaO_2_**	SB Healthy	25.5; 22.2-27.2	25.3; 20.5-30.6		22.7; 15.1-25.9
**mm Hg**	PEEP 5 Healthy	37.7; 31.0-39.2^1^	32.8; 29.8-36.8		32.5; 28.3-34.4^1^
				CPAP 5 LPS	13.5; 8.2-39.5
	PEEP 5 LPS	33.1; 24.3-37.0	24.3; 6.9-36.5^2^	PEEP 5 LPS	25.4; 8.7-32.1
				PEEP 15 LPS	32.3; 26.4-34.4*
**PaCO_2_**	SB Healthy	5.5; 4.7-5.9	5.0; 4.7-5.3		5.0; 4.5-5.2
**mm Hg**	PEEP 5 Healthy	4.9; 4.1-5.3^1^	4.8; 4.4-5.3		4.9; 4.4-5.3
				CPAP 5 LPS	6.7; 6.2-8.7^3^
	PEEP 5 LPS	4.7; 3.7-5.2	5.7; 4.4-6.6^2^	PEEP 5 LPS	5.5; 4.5-6.6^4^
				PEEP 15 LPS	5.1; 4.5-6.4^4^

(Peak airway pressure was not recorded in the healthy, spontaneously breathing pigs.) Note that the pigs given endotoxin (LPS) were all ventilated with PEEP 5 for the first 150 minutes (2.5 hours) and then either maintained on MV + PEEP 5 or switched to MV + PEEP 15 or SB + CPAP 5 for the next 2.5 hours (until 300 minutes); *n *= 6 in all groups. Significances (*P *< 0.05) after correction for multiple comparisons (Bonferroni):

Comparison with:

1: SB Healthy

2: Baseline

3: 150 minutes

4: CPAP 5 LPS

5: PEEP 5 LPS

*: indicates difference of borderline significance between MV+PEEP 15 and CPAP 5 (0.05 <*P *< 0.10; correction for multiple comparisons).

*Edema: *Abdominal edema formation, expressed as the normalized index (NI) for the whole 2D view of the abdomen, revealed no significant increase in spontaneously breathing piglets over the 5-hour study period (NI, 1.4; 1.0 to 2.5 (median and range) (NI at onset of the study period is always 1.0). In similarly healthy animals on MV with PEEP = 5, NI was 1.6; 1 to 3.3 (*P *< 0.05 compared with baseline).

*Inflammatory markers: *TNF-α and IL-6 concentrations in tissue samples from spontaneously breathing healthy controls were below detection limit in liver and displayed low levels also in the intestine (IL-6, 0.13; 0 to 0.52, TNF-α, 0.28; 0.19 to 0.44) and in the lung (IL-6, 0; 0 to 0.8, TNF-α, 2.16; 1.7 to 3.9; all values in pg/mg protein).

### Endotoxin-exposed pigs

*Gas exchange and hemodynamics: *Endotoxin infusion for 2.5 hours during MV+PEEP 5 increased airway and pulmonary artery pressures (MPAP) and PaCO_2 _and a decrease in PaO_2 _(Tables 1 and 2). Animals that continued on MV+PEEP 5 remained essentially stable in airway and vascular pressures but CO fell during the following 2.5 hours (total 5 hours). Animals that were switched to MV+PEEP 15 did not differ significantly from those that continued on MV+PEEP 5 more than in airway pressure. Animals switched to SB+CPAP 5 had lower airway pressures and an increased PaCO_2 _compared with the MV modes. PaO_2 _tended to be lower with SB+CPAP and highest with MV+PEEP 15. No changes in vascular pressures or CO (Table 2) were found. Intravenous maintenance fluid was given, with additional boluses to ameliorate a decrease in MAP (2.45; 1.7 to 3.5, 2.6; 2 to 3.3 and 2.35; 1.8 to 2.6 in the MV+PEEP5, MV+PEEP15, and SB+CPAP5 groups over the 5-hour study period, with no difference between groups), and MAP remained above 65 mm Hg throughout the experiment in all three groups. IAP increased in the LPS pigs and tended to be higher in the PEEP15 group than in the other two. APP decreased to <50 mm Hg in the PEEP15 group at the end of the study; see Table 2.

**Table 2:** Hemodynamics and abdominal pressures at baseline and at 150 and 300 minutes (median and range).

CO	SB Healthy				
**L/min**	**PEEP 5 Healthy**	**3.8; 2.2-4.8**	**3.7; 2.4-4.4**		**3.7; 2.7-5.1**
				CPAP 5 LPS	2.7; 1.9-3.0
	PEEP 5 LPS	2.6; 1.8-3.9	2.5; 1.7-5.3	PEEP 5 LPS	2.1; 1.8-2.9^3^
				PEEP 15 LPS	1.9; 1.5-2.7^4^
**HR**	SB Healthy	109.5; 95-123	97.5; 81-105		97.5; 89-103
bpm	PEEP 5 Healthy	107.0; 78-125	109.0; 76-132		107; 72-132
				CPAP 5 LPS	107.0; 93-117
	PEEP 5 LPS	88.2; 63-120	120.6; 87-146^2^	PEEP 5 LPS	105; 86-117
				PEEP 15 LPS	107.5; 95-127
**MAP**	SB Healthy	82.5; 59-92	67.5; 60-71		68.5; 58-70
mm Hg	PEEP 5 Healthy	72.5; 59-112	77.0; 56-103		83.0; 56-1111
				CPAP 5 LPS	88.5; 76-90
	PEEP 5 LPS	80.0; 57-115	74.2; 58-104	PEEP 5 LPS	81.0; 65-94
				PEEP 15 LPS	69.0; 59-78
**CVP**	SB Healthy				
mm Hg	PEEP 5 Healthy	8.5; 6-9	9; 5-10		8.5; 5-9
				CPAP 5 LPS	6.5; 5-9
	PEEP 5 LPS	7.5; 5-9	7.2; 4-11	PEEP 5 LPS	9.0; 3-11
				PEEP 15 LPS	9.5; 9-13^4^
**MPAP**	SB Healthy				
mm Hg	PEEP 5 Healthy	18.0; 17-24	17.5; 15-26		17.0; 16-25
				CPAP 5 LPS	34.5; 29-38
	PEEP 5 LPS	16.3; 14-21	37.1; 25-48^2^	PEEP 5 LPS	35.5; 25-45
				PEEP 15 LPS	33.5; 31-40
**APP**	SB Healthy				
mm Hg	PEEP 5 Healthy	60.8; 44.5-100	63; 42-86.5		70.3; 43.5-96
				CPAP 5 LPS	68.5; 62-77^3^
	PEEP 5 LPS	67.9; 37.5-69.5	55.7; 31-62	PEEP 5 LPS	63.8; 43-81
				PEEP 15 LPS	43.5; 31.6-65.0*
**IAP**	SB Healthy				
mm Hg	PEEP 5 Healthy	14.0; 5.5-18	13.5; 8-20		13.3; 8.5-21
				CPAP 5 LPS	17.5; 12-23.4
	PEEP 5 LPS	11.7; 5-25	19.0; 11-29^2^	PEEP 5 LPS	16.0; 10-22
				PEEP 15 LPS	22.5; 13-31.5

Note that the pigs given endotoxin (LPS) were all ventilated with PEEP 5 for the first 150 minutes (2.5 hours) and then either maintained on MV + PEEP 5 or switched to MV + PEEP 15 or SB + CPAP 5 for the next 2.5 hours (until 300 minutes); *n *= 6 in all groups. Significances (*P *< 0.05) after correction for multiple comparisons (Bonferroni):

Comparison with:

1: SB Healthy

2: Baseline

3: 150 minutes

4: CPAP 5 LPS

5: PEEP 5 LPS

*Difference of borderline significance between MV + PEEP 15 and CPAP 5 (0.05 <*P *< 0.10; correction for multiple comparisons).

**Figure 2:**
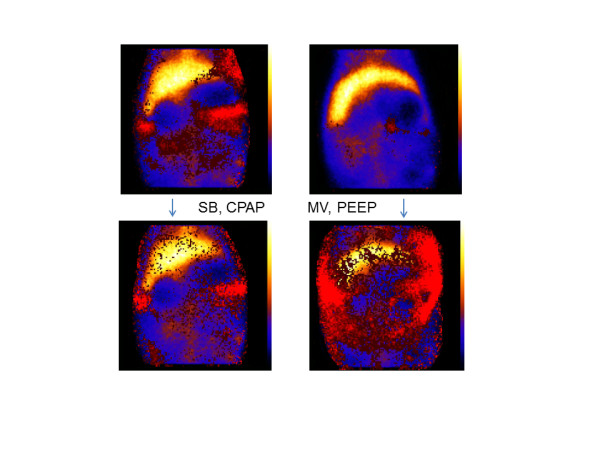
**Images of the abdomen showing the distribution of the normalized index (NI)**. The images have been constructed from the ^99m^Tc and ^111^In activity according to the NI equation in the Methods section. **(Upper panels) **Amount and distribution of edema in two animals after endotoxin infusion and 2.5 hours of mechanical ventilation with a PEEP of 5 cm H_2_O. **(Lower panels) **Edema in these animals after another 2.5 hours of either spontaneous breathing with a CPAP of 5 cm H_2_O or mechanical ventilation (MV) with a PEEP of 5 cm H_2_O. The red color indicates extravascular fluid. Note the larger increase in fluid in the animal on MV than on CPAP. The liver is shown in yellow in the upper part of the image.

*Edema: *NI had increased to 5.9; 4.9 to 6.9, after 5 hours of MV with PEEP 5 (*P *< 0.01 versus baseline) and was much higher than in healthy piglets on either SB or MV at 5 hours, as shown earlier. In animals that were switched from PEEP5 to MV+PEEP15 at 2.5 hours and followed up to 5 hours, a similar total increase (NI, 5.9; 5.5 to 6.5, *P *< 0.01 versus baseline) was observed. A smaller increase in edema formation was seen in the piglets that were switched after 2.5 hours on MV+PEEP 5 to SB+CPAP 5 for the following period up to 5 hours (NI, 4.7; 2.7 to 5.1, *P *< 0.05 versus MV with either 5 or 15 PEEP) (examples are shown in Figure 2).

**Figure 3:**
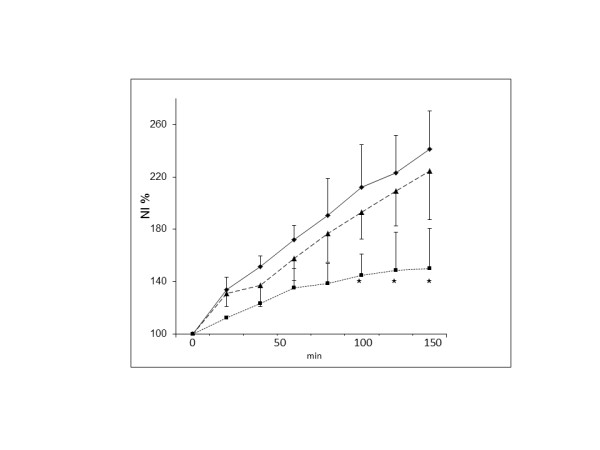
**Increase in edema (NI %) in the left-sided ROI, reflecting free fluid in the abdomen (ascites) during 2.5 hours of either (1) spontaneous breathing with a CPAP of 5 cm H_2_O (■), (2) mechanical ventilation with PEEP 5 cm H_2_O (▲) or (3) mechanical ventilation with PEEP 15 cm H_2_O (◆) in endotoxemic piglets**. All piglets had been on mechanical ventilation with PEEP 5 cm H_2_O for 2.5 hours before the allocation to one of the three groups. Note the 2- to 2.5-fold increase in edema over 150 minutes in the mechanically ventilated piglets, whereas the increase was only about 50% in the piglets on CPAP. The difference between CPAP and the two MV modes was significant during the latter part of the observation time (*P < 0.01 between either CPAP 5 and PEEP 5 or CPAP 5 and PEEP 15). No significant difference was seen between PEEP 5 and PEEP 15.

Similar increases were established in the ROI corresponding to the left aspects of the abdomen, reflecting, to a major extent, abdominal free fluid (ascites). This is illustrated in Figure 3, with the amount of edema set as 100% at onset of either ventilatory mode after the preceding 2.5 hours of MV with PEEP 5 and endotoxin infusion. As can be seen, PEEP 15 caused a 2.5 times increase in edema formation, and PEEP5, almost as much as PEEP 15. Piglets on SB with CPAP 5 showed only 50% increase in edema formation.

The ROI corresponding to the liver showed a fivefold increase in NI compared with baseline (NI, 4.9; 3.1 to 5.8; *P *< 0.01 for all three groups pooled). No significant difference was seen between groups.

*Inflammatory markers: *TNF-α and IL-6 concentrations in liver and intestinal tissue samples were elevated in the pigs ventilated with PEEP 5 or 15 cm H_2_O, with no difference between them (median values between 2 and 5 pg/mg protein) (Table 3). The endotoxin-exposed spontaneously breathing piglets showed much lower concentrations (median values between 1 and 2 pg/mg protein).

**Table 3:** Inflammatory markers in the endotoxin-exposed piglets (median and range) (pg/mg protein, except plasma where pg/mg)

	Liver		Intestine		Lung		Plasma	
Median (range)	IL-6	TNFα	IL-6	TNFα	IL-6	TNFα	IL-6	TNFα
CPAP 5	2.06 (1.7-2.4)	1.67* (1.7-2.5)	1.91* (1.1-2.3)	1.10 (0.8-1.6)	20.65 (12.9-34.7)	37.1 (31.1-45.9)	1526 (1526-2500)	566.0 (410-1132)
PEEP 5	5.53 (1.8-9.3)	5.18 (2.4-9.3)	6.21 (2.2-7.8)	2.70 (1.0-4.8)	34.2 (9.2-42.4)	43.9 (22.1-46.6)	2500 (1104-2500)	590.3 (288-724)
PEEP 15	2.87 (1.8-9.7)	3.88 (2.2-8.1)	3.23 (1.2-6.4)	2.24 (1.5-2.7)	23.1 (7.7-37.8)	44.1 (31.4-48.0)	953.9 (445-2500)	482.6 (291-818)

^a^Significantly different from PEEP.

Lung-tissue concentrations of TNF-α and IL-6 were similar in all three endotoxin-exposed groups (Table 3) and markedly elevated compared with the healthy spontaneously breathing control animals. Plasma concentrations of TNF-α and IL-6 were also similar in the three endotoxin-exposed groups

**Figure 4:**
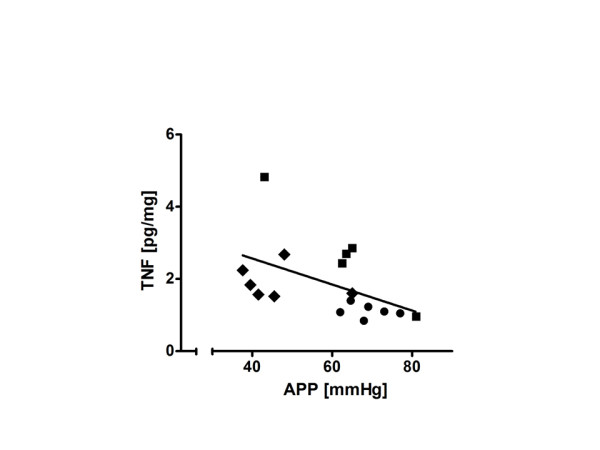
**Correlation between abdominal perfusion pressure (APP; arterial mean pressure minus bladder pressure) and TNF-α in intestine**. Data have been pooled from the SB CPAP 5 group (●), the MV PEEP 5 group (■), and the MV PEEP 15 group (♦) (*r *= 0.61; *P *= 0.01). Moreover, the two groups, CPAP and MV 15, were significantly different in both APP and TNF-α (*P *< 0.01 for both). See also text.

*Correlation tests: *Abdominal edema increased with decreasing abdominal perfusion pressure (APP; mean arterial minus intraabdominal pressure) in the piglets on MV with PEEP 15 (r, 0.52; *P *= 0.05) but not in piglets on CPAP or MV PEEP 5. No significant correlations were seen between NI and a number of pressure variables (MAP, MPAP, CVP, IAP), nor with pH, BE, PaO_2_, or PaCO_2_. TNF-α in intestine increased with decreasing APP (Figure 4). As can be seen in Figure 4, piglets on PEEP 15 had a lower APP and higher TNF-α than did the CPAP piglets, whereas PEEP 5 piglets were distributed along the whole range of APP and TNF-α values (*r *= -0.61; *P *= 0.01 for pooled data from all three groups).

## Discussion

We showed that abdominal edema and inflammatory markers in intestine and liver are increased by mechanical ventilation *per se *compared with spontaneous breathing in endotoxemic piglets.

In a previous study, we analyzed the impact of mechanical ventilation on abdominal lymph flow in the same sepsis-like model as in the present study [8]. The results showed that the abdominal lymph production increased with mechanical ventilation compared with spontaneous breathing with CPAP. At the same time, the lymph flow from the abdomen, passing through the thorax in the thoracic duct to major veins, was decreased. The increased lymph production was reasonably explained by increased capillary pressure promoting extravasation of fluid, whereas impeded drainage could be explained by compression of the thoracic duct by increased intrathoracic pressure. In the present study, we showed that the net result is abdominal fluid accumulation.

Not only was there an increased amount of ascites, but an increase of liver and intestinal edema also was seen with the gamma camera measurements (liver) and by visual inspection of the organs after termination of the experiment. We took intestinal tissue samples in a previous study, in which we validated the technique of assessing edema by calculating the Normalized Index (NI). We saw how lymph vessels in intestinal villi were more frequent and wider, as were also the villi themselves, in the septic animal than in the healthy control [9]. Similar findings have been made previously (for example, by Schoots and co-workers, although they were not specifically referred to in their article [12]. It should be made clear that the recording of liver edema by NI is very rough and can serve only as an indicator that edema is produced also within this organ.

The common denominator for increased capillary leakage and impeded lymph drainage is proposed to be increased intrathoracic pressure. This suggests that any ventilatory mode that lowers airway and intrathoracic pressure may also be beneficial for the abdomen. "Lung-protective" ventilation with low tidal volumes has emerged as an important treatment modality in acute lung injury and ARDS [13], and a consequence by applying lung-protective ventilation is in general a reduced intrathoracic pressure. We used a tidal volume of 10 ml/kg, which has been ordinary in healthy or non-ARDS lungs [14], but it was recently shown that tidal volumes of 6 or up to 8 ml/kg also result in better clinical outcome in surgical patients with no preexisting lung disease [15]. One may ask, to what extent have low tidal volumes during ventilator treatment improved outcome by lung protection or by abdomen protection. However, it cannot be excluded that other mechanisms than intrathoracic pressure act in improving abdominal fluid balance; the small difference between PEEP 5 and 15 may indicate this.

Obviously, more remains to be studied regarding an optimal ventilator setting for both lung and abdomen.

An increase in the concentrations of TNF-α and IL-6 was found in the intestine and liver with mechanical ventilation and much less so with spontaneous breathing and CPAP. Previous studies showed increased expression of proinflammatory cytokines in abdominal organs in animals exposed to injurious mechanical ventilation [16]. In the present study, we also saw an inflammatory response during "conventional" mechanical ventilation, with no intention to make the ventilation "injurious." During spontaneous breathing with CPAP of 5 cm H_2_O, much less inflammation and edema were seen. Moreover, lung and plasma concentrations of the inflammatory markers did not differ between mechanical ventilation and spontaneous breathing, suggesting that the different concentrations in the abdominal organs were not caused by a spread from the lung or other extraabdominal organs but rather reflect different degrees of tissue synthesis in the intestine and the liver.

The plasma concentrations of the inflammatory markers were higher than in the abdominal organs. However, the animals had been exposed to intravenous endotoxin infusion that must have provoked an inflammatory response in different circulating cells in the immune system. Moreover, quantitative differences in plasma and tissue concentrations cannot be interpreted as similar quantitative differences in inflammatory response.

We chose TNF-α and IL-6 as markers of inflammation, and one may ask whether other markers would have given another result. However, Pierrakos and Vincent [17] reviewed a large number of biomarkers and concluded that no one special marker/entity has yet been proven in experimental and clinical studies, to be of more relevance and specificity than the others.

Correlations were seen between abdominal perfusion pressure on the one hand and abdominal edema and TNF-α in intestine on the other. Edema is a hallmark of inflammation, but one may speculate that edema can also cause inflammation. Interactions between vascular permeability and inflammation were discussed in a recent review on the procoagulant and proinflammatory plasma contact system [18], and in high-altitude pulmonary edema, inflammation appears to be a late effect of the edema [19-21]. In a review [22], it is concluded that "although high altitude pulmonary edema develops in the absence of any local or systemic inflammation, inflammatory activation occurs in later stages". To what extent it can explain the inflammation in the present study certainly requires more investigation but remains an interesting possibility. Our finding that low abdominal perfusion pressure was accompanied by increased inflammatory marker concentration may suggest another, parallel, mechanism of inflammation, or the lowered perfusion pressure may be a consequence of the edema. Lowered perfusion pressure can disturb cellular metabolism that triggers an inflammatory response [23-25]. It may be emphasized that the correlation between abdominal perfusion pressure and inflammatory markers was seen although MAP was kept above 65 mm Hg and thus in accordance with the guidelines in the Surviving Sepsis Campaign [26].

In a comprehensive review, Vollmar and Menger [27] listed a number of diagnoses that are all at risk of intestinal ischemia, with sepsis as one important factor [27]. They also emphasized that intestinal ischemia and reperfusion may be more dangerous than ischemia alone. Parks and Granger [28] showed that the injury observed after 3 hours of ischemia and 1 hour of reperfusion of the small intestine is more severe than that observed after 4 hours of ischemia. Thus, some reaction initiated by the return of oxygenated blood to the ischemic intestine is one cause of the reperfusion-induced injury [29].

In our study, reperfusion has not been a deliberate part of the protocol and must have been of limited importance when interpreting our results. It may rather be that ischemia plus reperfusion might make the conditions we studied even worse.

## Conclusions

In summary, we have shown, in a sepsis-like porcine model, that mechanical ventilation increases abdominal edema and inflammation in intestine and liver. These changes were much smaller during spontaneous breathing. Mechanical ventilation has been discussed as a contributing factor to multiple-system organ failure [30]. In view of the frequent cause of death by abdominal organ failure in sepsis and even in acute respiratory distress syndrome [31,32], potential negative effects by mechanical ventilation *per se *should be considered.

## Key messages

• Experimental endotoxemia, mimicking sepsis, caused abdominal free fluid (ascites) and abdominal organ edema.

• The experimental endotoxemia caused increased concentrations of inflammatory markers in liver and intestine.

• Mechanical ventilation with PEEP 5 or 15 cm H_2_O caused more abdominal edema and inflammation than spontaneous breathing with CPAP of 5 cm H_2_O.

• A likely mechanism of the increased abdominal edema and, possibly, inflammation is increased intrathoracic pressure that increases capillary pressure (increased leakage) and obstructs the thoracic duct (impeded lymph drainage).

## Abbreviations

APP: abdominal perfusion pressure; BE: base excess; CO: cardiac output; CPAP: continuous positive airway pressure; CVP: central venous pressure; HR: heart rate; IAP: intraabdominal pressure; IL-6: interleukin 6; In (^111^In): radioactive: gamma-emitting indium; KCl: potassium chloride; LPS: lipopolysaccharide; MAP: mean arterial pressure; MPAP: mean pulmonary artery pressure; MV: mechanical ventilation; NI: normalized index; PaO_2_: arterial oxygen tension; PaCO_2_: arterial carbon dioxide tension; P_aw _max: peak airway pressure; P_aw _ei: end-inspiratory airway pressure; PEEP: positive end-expiratory pressure; RBC: red blood cell; ROI: region of interest; SB: spontaneous breathing; Tc (^99m^Tc): radioactive, gamma-emitting technetium; TNF-α: tumor necrosis factor α.

## Competing interests

The authors declare that they have no competing interests.

## Authors' contributions

ML designed the study (together with GH), performed the animal experiments, and analyzed the physiological data and (together with EM) the gamma camera results. MB carried out the analysis of the inflammatory markers. EM made the gamma camera measurements and made dedicated software for the analysis. GH designed and supervised the study and the analysis of results. All authors (ML, MB, EM, GH) contributed to the drafting of the manuscript.
